# Physical activity and clustered cardiovascular disease risk factors in young children: a cross-sectional study (the IDEFICS study)

**DOI:** 10.1186/1741-7015-11-172

**Published:** 2013-07-30

**Authors:** David Jiménez-Pavón, Kenn Konstabel, Patrick Bergman, Wolfgang Ahrens, Hermann Pohlabeln, Charalampos Hadjigeorgiou, Alfonso Siani, Licia Iacoviello, Dénes Molnár, Stefaan De Henauw, Yannis Pitsiladis, Luis A Moreno

**Affiliations:** 1 Department of Physiotherapy and Nursing, School of Health Sciences, University of Zaragoza, Zaragoza, Spain; 2 GENUD: (Growth, Exercise, NUtrition and Development) Research Group, Department of Physiotherapy and Nursing, Faculty of Health Sciences, University of Zaragoza, Avd. Domingo Miral s/n, 50009, Zaragoza, Spain; 3 Research Centre National Institute for Health Development, Tallinn, Estonia; 4 Department of Sport Sciences, Linnaeus University, Kalmar, Sweden; 5 Bremen Institute for Preventive Research and Social Medicine, University of Bremen, Bremen, Germany; 6 Research & Education Institute of Child Health, Strovolos, Cyprus; 7 National Research Council, Institute of Food Sciences (ISA-CNR), Unit of Epidemiology and Population Genetics, Avellino, Italy; 8 Fondazione di Ricerca e Cura ‘Giovanni Paolo II’, Università Cattolica del Sacro Cuore, Campobasso, Italy; 9 Department of Paediatrics, University of Pécs, Pécs, Hungary; 10 Department of Public Health, Gent University, Ghent, Belgium; 11 Institute of Cardiovascular & Medical Sciences, University of Glasgow, Glasgow, UK

**Keywords:** Accelerometers, Cardiovascular disease risk factors, Physical activity, Younger children

## Abstract

**Background:**

The relevance of physical activity (PA) for combating cardiovascular disease (CVD) risk in children has been highlighted, but to date there has been no large-scale study analyzing that association in children aged ≤9 years of age. This study sought to evaluate the associations between objectively-measured PA and clustered CVD risk factors in a large sample of European children, and to provide evidence for gender-specific recommendations of PA.

**Methods:**

Cross-sectional data from a longitudinal study in 16,224 children aged 2 to 9 were collected. Of these, 3,120 (1,016 between 2 to 6 years, 2,104 between 6 to 9 years) had sufficient data for inclusion in the current analyses. Two different age-specific and gender-specific clustered CVD risk scores associated with PA were determined. First, a CVD risk factor (CRF) continuous score was computed using the following variables: systolic blood pressure (SBP), total triglycerides (TG), total cholesterol (TC)/high-density lipoprotein cholesterol (HDL-c) ratio, homeostasis model assessment of insulin resistance (HOMA-IR), and sum of two skinfolds (score CRFs). Secondly, another CVD risk score was obtained for older children containing the score CRFs + the cardiorespiratory fitness variable (termed score CRFs + fit). Data used in the current analysis were derived from the IDEFICS (‘Identification and prevention of Dietary- and lifestyle-induced health EFfects In Children and infantS’) study.

**Results:**

In boys <6 years, the odds ratios (OR) for CVD risk were elevated in the least active quintile of PA (OR: 2.58) compared with the most active quintile as well as the second quintile for vigorous PA (OR: 2.91). Compared with the most active quintile, older children in the first, second and third quintiles had OR for CVD risk score CRFs + fit ranging from OR 2.69 to 5.40 in boys, and from OR 2.85 to 7.05 in girls.

**Conclusions:**

PA is important to protect against clustering of CVD risk factors in young children, being more consistent in those older than 6 years. Healthcare professionals should recommend around 60 and 85 min/day of moderate-to-vigorous PA, including 20 min/day of vigorous PA.

Please see related commentary: http://www.biomedcentral.com/1741-7015/11/173.

## Background

The risk for future cardiovascular disease (CVD) in apparently healthy children can be assessed by a clustering of individual risk factors in the same individual, as it describes a status with several of these risk factors being high simultaneously [1]. A risk factor understands as a key parameter that can model the risk for CVD. Obesity is one of the main risk factors associated with increased CVD risk in children and adolescents [2]. Specifically, conditions such as overweight and obesity in children have, in recent years, reached epidemic proportions and they are still rapidly increasing, with marked effects at all socioeconomic levels and across ethnicities [3-5]. Recently, several reviews have highlighted the relevance of physical activity (PA) as the main therapeutic tool for combating CVD risk in children and adolescents [6-8]. The use of objectively-measured PA as well as the use of clusters of metabolic risk have been suggested as being appropriate for more precise analysis of these relationships [6]. Several studies have shown an inverse relationship between objective PA and metabolic syndrome risk factors in healthy children and adolescents [9-16]. Most of the studies analyzing the association between PA and CVD risk are based on children from different parts of the European Youth Heart Study, with children of 9 and 15 years of age [9,10,12-14,16]. All of these studies observed an inverse relationship. Recently, this inverse association has also been observed in a relatively small study (n = 223) performed with Swedish children aged 8 to 11 years [15]. Only one cross-sectional study has addressed these relationships in children aged <9 years, in particular in younger children (<6 years) [11]. Butte *et al*. studied a sample of 897 Hispanic children (4 to 19 years) in the USA; they observed no association of PA with the presence of CVD risk, but a significant association was found with the number of components included in the concept of metabolic syndrome (from 0 to 5 components such as high waist circumference, lower high-density lipoprotein cholesterol (HDL-c), higher levels of hypertriglyceridemia, high blood pressure, and fasting glucose) [11]. Finally, the group of Andersen *et al*. failed to find any association at age of 6 years (n = 435), although concluded that a clustering of CVD risk factors developed between the age of 6 and 9 years [1,17]. To the best of our knowledge, there has not been any study analyzing the association of objectively-measured PA in relation to clustered CVD risk factors focusing on children aged from 2 to 9 years, with gender-specific information, in a relatively large sample.

Since 2000, there have been several PA recommendations with respect to duration and intensity required to ensure a healthy lifestyle in children and adolescents (age range: 6 to 17 years) [9,18-23] and movement coordination benefits in younger children (age range: 2 to 5 years) [24]. The consensus recommendation has been that children and adolescents should participate in ≥60 minutes of PA of moderate to vigorous intensity daily (or most days of the week) [18-23]. Later, in 2006, Andersen *et al*. recommended daily PA of 90 minutes duration based on their findings in children aged 9 and 15 years [9]. Conversely, Wittmeier *et al*. suggested 60 minutes per day instead of 90 minutes in children aged 8 to 11 years as an attainable goal in view of the lower percentage of those achieving higher intensity activity in their study [18]. Finally, in 2007 a recommendation of 60 minutes of PA was made for young children (2 to 5 years) based on evidence of benefits accruing with respect to cognitive performance and motor skills. However, there was not enough evidence to suggest metabolic improvement [24]. Hence, more specific recommendations on PA (levels and intensities) for health benefits are needed, especially in relation to age groups (including those <6 years) and/or gender.

The objectives of the present study were: (1) to evaluate the associations between objectively-measured PA intensities and clustered CVD risk factors in a large sample of European children aged 2 to 9 years, and (2) to provide evidence for the development of gender-specific recommendations of PA for this young population.

## Methods

### Study population

Data used in the current analysis were derived from the IDEFICS (‘Identification and prevention of Dietary- and lifestyle-induced health EFfects In Children and infantS’) study. A total of 16,224 children aged 2 to 9 years were recruited during the baseline survey, which was conducted between 2007 and 2008 in 8 European countries (Italy, Estonia, Cyprus, Belgium, Sweden, Germany, Hungary, Spain) [25]. All participants met the general IDEFICS inclusion criteria: age group 2 to 9 years, available data on body mass and height, and completion of the parental questionnaire. From the total sample of 16,224 children, a subset of 12,134 had valid data for age, body mass, height, body mass index (BMI) and blood sample parameters. As accelerometry was measured only in a subset from every center due to availability of accelerometers, when the objective measurement of PA was included in the analyses the sample size was reduced. For the purposes of the current analyses, only subjects (n = 3,019) with a complete set of data that included total triglycerides (TG), total cholesterol (TC), HDL-c, glucose, insulin, systolic blood pressure (SBP), sum of two skinfold thickness measurements, exposure (PA intensities) and confounding variables were included. No differences with respect to mean age, body mass and Z score BMI were observed between individuals in the subset with complete data and the rest of the sample. The study was conducted according to the standards of the Declaration of Helsinki. (Edinburgh 2000 revision), the Good Clinical Practice, and the legislation about clinical research in humans. All applicable institutional and governmental regulations pertaining to the ethical use of human volunteers were followed during this research. Approval by the appropriate ethics committees was obtained by each of the eight participating centers carrying out the fieldwork (Belgium: Ethics Committee, University Hospital, Gent; Cyprus: Cyprus National Bioethics Committee; Estonia: Tallinn Medical Research Ethics Committee; Germany: Ethics Committee, University of Bremen; Hungary: Egészségügyi Tudományos Tanács, Pécs; Italy: Comitato Etico, ASL Avellino; Spain: Comité Ético de Investigación, Clínica de Aragón (CEICA); Sweden: Regional Ethics Review Board, University of Gothenburg). Written informed consent was obtained from the parents (or guardian) of each child participating in the study.

### Measurements

For quality management, all measurements followed detailed standard operating procedures that were laid down in the general survey manual and finalized after the pretest of all survey modules [26]. Field personnel from each study center participated in the central training and organized local training sessions thereafter. The coordinating center conducted site visits to each study location during both field surveys to check adherence of field [25].

### Socioeconomic status (SES)

SES was estimated using the International Standard Classification of Education. A score was calculated from the highest education and qualification levels of both parents. Five groups were defined using a scale from 0 to 6: level 1 (0 and 1), level 2 (2), level 3 (3), level 4 (4), and level 5 (5 and 6); the lower the score, the lower SES.

### Physical examinations

Body mass was measured in light clothing to the nearest 0.1 kg with an electronic scale (TANITA BC 420 SMA, Tokyo, Japan). Height was measured without shoes to the nearest 0.1 cm using a stadiometer (Seca 225; Seca, Hamburg, Germany). Skinfold thicknesses were measured with a Holtain caliper (Holtain Ltd., Croswell, UK) at the triceps and subscapular sites. Blood pressure was measured with an electronic sphygmomanometer (Welch Allyn 4200B-E2; Welch Allyn, Aston Abbotts, UK) [27] preferably in the right arm with the child seated and in a calm environment. Two measurements were taken at 2-minute intervals and, if they differed by >5%, a third measurement was taken. The mean of the two (or three) measurements was used in all statistical analyses.

### Physical activity

The uniaxial Actigraph accelerometer (Actigraph MTI, model GT1M; Manufacturing Technology Inc., Fort Walton Beach, FL, USA) and the ActiTrainer (http://www.actitrainer.com) were used to measure PA. The ActiTrainer technology is based on the ActiGraph accelerometer with additional functions (heart rate). The rationale to use the ActiGraph in younger and ActiTrainer in older children was to record, when possible, the heart rate. However, in the current study only data from accelerometers were used and crossvalidation was not necessary as both accelerometers are essentially the same model of ActiGraph. Prior to data collection, parents were instructed in the correct positioning of the accelerometer; that is, to attach the accelerometer to the right hip of the child during their waking day by means of an elastic belt adjusted to ensure close contact with the body. The accelerometer needed to be worn all day over 4 to 5 days, except during water-based activities and during sleep. Recordings were for at least 6 h/day for at least 3 days (2 weekdays and 1 day of the weekend or holiday) in accord with the results of the reliability analysis indicating a minimum duration of 6 h per day of monitoring to achieve 80% reliability [28]. The sampling interval (epoch) was set at 15 s. Non-wear time was excluded from the data by means of an automated method that uses an algorithm developed using R (version R 2.9.0.; R Foundation for Statistical Computing, Vienna, Austria; http://www.R-project.org). Thus, periods of 20 minutes or more consecutive zero counts were replaced by missing data code before further analysis [28]. A measure of average total volume of activity (hereafter called total PA) was expressed as the sum of recorded counts divided by total daily registered time expressed in minutes (counts/minute; cpm). The cut-offs to define the PA intensity categories were derived from previously-validated cut-offs [29], with time spent in light PA (minutes) defined as the sum of time-per-day in which counts per epoch were 26 to 573 cpm. The time engaged in moderate PA was calculated based upon a cut-off of 574 to 1,002 cpm per epoch. The time engaged in vigorous PA was calculated based upon a cut-off of ≥1,003 cpm per epoch. In addition, the time spent at the ‘effective’ intensity level was calculated as the sum of time spent in moderate + vigorous PA (MVPA).

### Cardiorespiratory fitness

Fitness was measured by the progressive 20-m shuttle run test [30]. This test required subjects to run back and forth between two lines set 20 m apart at a pace determined by audio signals. The initial speed was set at 8.5 km/h increasing by 0.5 km/h every minute (1 minute equals 1 stage). The test was completed when the child failed to reach the end lines in time with the audio signals on two consecutive occasions. The final score was computed as the number of stages completed (precision of 0.5 stages). Stages completed were used to estimate the VO_2max_[30].

### Biological samples

A detailed description of the blood sampling procedures has been published elsewhere [31]. Briefly, blood samples were obtained after an overnight fast and previous confirmation by questionnaire of achievement this criterion. Blood glucose, TC, HDL-c and TG were assessed on site at each study center by point-of-care analysis using a Cholestech LDX analyzer (Cholestech, Hayward, CA, USA) [32]. Serum insulin concentrations were determined by luminescence immunoassay in a central laboratory using an AUTO-GA Immulite 2000, Siemens, Eschborn, Germany. To derive a measure of insulin resistance we used the homeostasis model assessment (HOMA-IR) [33] using fasting glucose and plasma insulin according to the following formula: HOMA-IR = [fasting insulin (pmol/l)/6.945] × [fasting glucose (mmol/l)/22.5].

### Cardiovascular risk score

According to Andersen *et al*. [9] a continuous score clustering CVD risk factors (CRFs) was computed using the following variables: SBP, TG, TC/HDL-c ratio, HOMA-IR, and sum of two skinfolds (score CRFs). Since the 20-m shuttle run test was only performed in children >6 years of age, a second CVD risk score was obtained for older children containing the score CRFs + the cardiorespiratory fitness variable using the total number of stages (termed score CRFs + fit). Z scores were calculated for each risk factor variable by age and gender, followed by a summing of individual Z scores to create the two clustered risk scores. Cardiorespiratory fitness Z score was multiplied by -1 to indicate higher metabolic risk with increasing value. The lower the CVD risk the better the overall CVD risk factor profile.

### Statistical analysis

Predictive Analytics SoftWare (PASW, version 18; SPSS Inc., Chicago, IL, USA) was used to perform the analyses. Statistical significance was set at *P* <0.05. The data are presented as mean ± standard deviation (SD) unless otherwise stated. Mean and SD for CVD risk were calculated for age and gender groupings of the children who had a complete set of measurements. Age groups were recorded as younger children (between 2 to 6 years) and older children (between 6 to 9 years). The distributions of PA were observed to be skewed and so to achieve normality of distributions, moderate PA, vigorous PA, and MVPA were transformed to the natural logarithm values. Individuals >1 SD away from the mean in the clustered risk scores were defined as being ‘at risk’. For descriptive variables, the Student’s t test was used to test the differences between genders. To examine the association between PA intensities and CVD risk scores, partial correlation analyses adjusted for country were conducted in both age groups.

Age and gender-specific quintiles were created for each PA intensity. One-way analysis of covariance (ANCOVA) was used to test the differences in CVD risk scores (dependent variables) among quintiles of PA (fixed factor) segregated by age and gender and adjusted for country (dummy variable) and SES.

Logistic regression models were used to calculate the odds ratios (OR) for having clustered risk score (dichotomous variable; Z score above 1 SD) across quintiles of different PA intensities (quintile 5 as reference) segregated by age and gender. Country (dummy variable) and SES were included as covariates. Moreover, descriptive analyses were performed to stand out the mean, SD and range of time corresponding at each quintile among the different PA intensities segregated by age and gender. Finally, the mean, SD and range at the highest quintile (Q5) of PA was selected as potential recommendation.

## Results

### Descriptive characteristic of the study sample

Table 1 summarizes the descriptive characteristics of the study sample. In younger children, girls had significantly higher sum of two skinfolds, insulin and HOMA-IR values than boys (all *P* <0.001), while the boys had higher weight, height, glucose, HDL-c and all PA intensities (except vigorous PA) than girls (all *P* <0.05). Age, BMI, SBP, diastolic blood pressure (DBP), TC, TG, vigorous PA and CVD risk score CRF mean values were similar between genders. In older children, girls had significantly higher sum of two skinfolds, insulin, HOMA-IR, TC and TG values than boys (all *P* <0.01), while boys had greater height, SBP, glucose, HDL-c, moderate, vigorous, MVPA and total PA as well as cardiorespiratory fitness than their female counterparts (all *P* <0.05). The means of age, weight, BMI and CVD risk scores (CRFs and CRFs + fit) were similar in both genders. When the Bonferroni correction factor for multiple tests was applied, only those with *P* <0.0025 remained significant.

**Table 1:** Descriptive characteristics of the study participants

**Characteristic**	**All**	**Boys**	**Girls**	***P *value**
Group 2 to 6 years	n = 994	n = 524	n = 470	
Age, years	4.4 ± 0.8	4.5 ± 0.8	4.4 ± 0.8	0.226
Body mass, kg	18.3 ± 3.5	18.5 ± 3.5	18.0 ± 3.4	0.011
Height, cm	107.2 ± 7.4	107.8 ± 7.4	106.6 ± 7.5	0.011
BMI, kg/m^2a^	15.8 ± 1.7	15.9 ± 1.6	15.7 ± 1.8	0.170
Sum of two skinfolds, mm^a^	16.5 ± 5.0	15.3 ±54.3	17.7 ± 5.3	<0.001^b^
DBP, mmHg	62.1 ± 6.2	62.0 ± 6.1	62.2 ± 6.3	0.617
SBP, mmHg	97.4 ± 8.4	97.7 ± 8.5	97.1 ± 8.2	0.238
Glucose, mmol/l	4.51 ± 0.51	4.55 ± 0.54	4.46 ± 0.48	0.010
Insulin, pmol/l^a^	22.64 ± 18.47	21.39 ± 17.36	24.10 ± 19.52	0.011
HOMA-IR^a^	0.68 ± 0.62	0.65 ± 0.57	0.72 ± 0.66	0.011
Cholesterol, mmol/l	4.02 ± 0.73	3.99 ± 0.72	4.06 ± 0.74	0.127
HDL-c, mmol/l	1.23 ± 0.34	1.29 ± 0.33	1.19 ± 0.35	0.001^b^
Triglycerides, mmol/l	0.46 ± 0.25	0.45 ± 0.23	0.48 ± 0.28	0.188
Light PA, min/day^a^	395 ± 65	400 ± 65	390 ± 66	0.019
Moderate PA, min/day^a^	31 ± 16	34 ± 17	27 ± 14	<0.001^b^
Vigorous PA, min/day^a^	5 ± 5	6 ± 6	5 ± 5	0.281
MVPA, min/day^a^	36 ± 20	39 ± 21	32 ± 17	<0.001^b^
Total PA, cpm	598 ± 174	627 ± 179	565 ± 162	<0.001^b^
CVD risk score CRFs	0.02 ± 2.74	0.01 ± 2.70	0.03 ± 2.77	0.879
Group 6 to 9 years	n = 2,025	n = 1,038	n = 987	
Age, years	7.6 ± 0.8	7.6 ± 0.8	7.6 ± 0.8	0.926
Body mass, kg	27.7 ± 6.7	28.0 ± 6.9	27.5 ± 6.3	0.088
Height, cm	127.5 ± 7.3	128.2 ± 7.4	126.9 ± 7.3	<0.001^b^
BMI, kg/m^2a^	16.9 ± 2.9	16.9 ± 3.0	16.9 ± 2.8	0.618
Sum of two skinfolds, mm^a^	19.8 ± 9.5	18.3 ± 9.2	21.4 ± 9.2	0.070
DBP, mmHg	64.2 ± 6.5	64.0 ± 6.7	64.5 ± 6.3	0.018
SBP, mmHg	103.2 ± 8.7	103.7 ± 8.7	102.8 ± 8.6	0.070
Glucose: mmol/l	4.81 ± 0.52	4.88 ± 0.52	4.75 ± 0.49	<0.001^b^
Insulin, pmol/l^a^	35.84 ± 24.65	33.41 ± 22.43	38.34 ± 24.93	<0.001^b^
HOMA-IR^a^	1.13 ± 0.83	1.07 ± 0.77	1.20 ± 0.84	<0.001^b^
Cholesterol, mmol/l	4.19 ± 0.83	4.11 ± 0.77	4.28 ± 0.84	<0.001^b^
HDL-c, mmol/l	1.40 ± 0.38	1.42 ± 0.39	1.38 ± 0.37	0.016
Triglycerides, mmol/l	0.47 ± 0.26	0.45 ± 0.24	0.49 ± 0.29	<0.001^b^
Light PA, min/day^a^	364 ± 64	364 ± 63	364 ± 17	0.940
Moderate PA, min/day^a^	35 ± 17	41 ± 19	30 ± 12	<0.001^b^
Vigorous PA, min/day^a^	8 ± 6	8 ± 6	7 ± 8	0.001^b^
MVPA, min/day^a^	43 ± 22	49 ± 23	37 ± 18	<0.001^b^
Total PA, cpm	581 ± 169	609 ± 175	553 ± 157	<0.001^b^
Cardiorespiratory fitness, ml/kg/min^c^	41.7 ± 14.5	42.2 ± 14.3	41.3 ± 13.8	0.01
Stages (total number)	1.9 ± 1.3	2.1 ± 1.5	1.7 ± 1.1	<0.001^b^
CVD risk score CRFs	0.13 ± 3.11	0.09 ± 3.03	0.18 ± 3.19	0.501
CVD risk score CRFs + fit^c^	−0.28 ± 3.26	−0.26 ± 3.016	−0.31 ± 3.36	0.775

CVD risk score CRFs includes SBP, HOMA-IR, ratio cholesterol/HDL-c, triglycerides and sum of two skinfolds; CVD risk score CRFs + fit added cardiorespiratory fitness.

^a^Non-transformed data are presented in this table but analyses were performed on log-transformed data.

^b^Significant differences after to apply the Bonferroni correction factor for multiple tests.

^c^Sample size including cardiorespiratory fitness was 835 participants (414 boys).

*BMI* body mass index, *CRF* CVD risk factor, *CVD* cardiovascular disease, *DBP* diastolic blood pressure, *HDL* high-density lipoprotein, *HOMA*-*IR* homeostasis model assessment, *MVPA* moderate + vigorous PA, *PA* physical activity, *SBP* systolic blood pressure.

### Correlations

Partial correlations between PA intensities and CVD risk scores (CRFs and CRFs + fit) in both age groups were investigated following adjustment for country. In young children, only vigorous PA was inversely correlated with the CVD risk score CRFs (r: -0.086; *P* <0.01; n = 994). In older children, moderate PA, vigorous PA, MVPA and total PA intensities were inversely correlated with both CVD risk scores (score CRFs ranged from -0.089 to -0.166, all *P* <0.01, n = 2,025; score CRFs + fit ranged from -0.111 to -0.251, all *P* <0.001, n = 835).

### Differences in mean Z score by quintiles of PA

**Figure 1:**
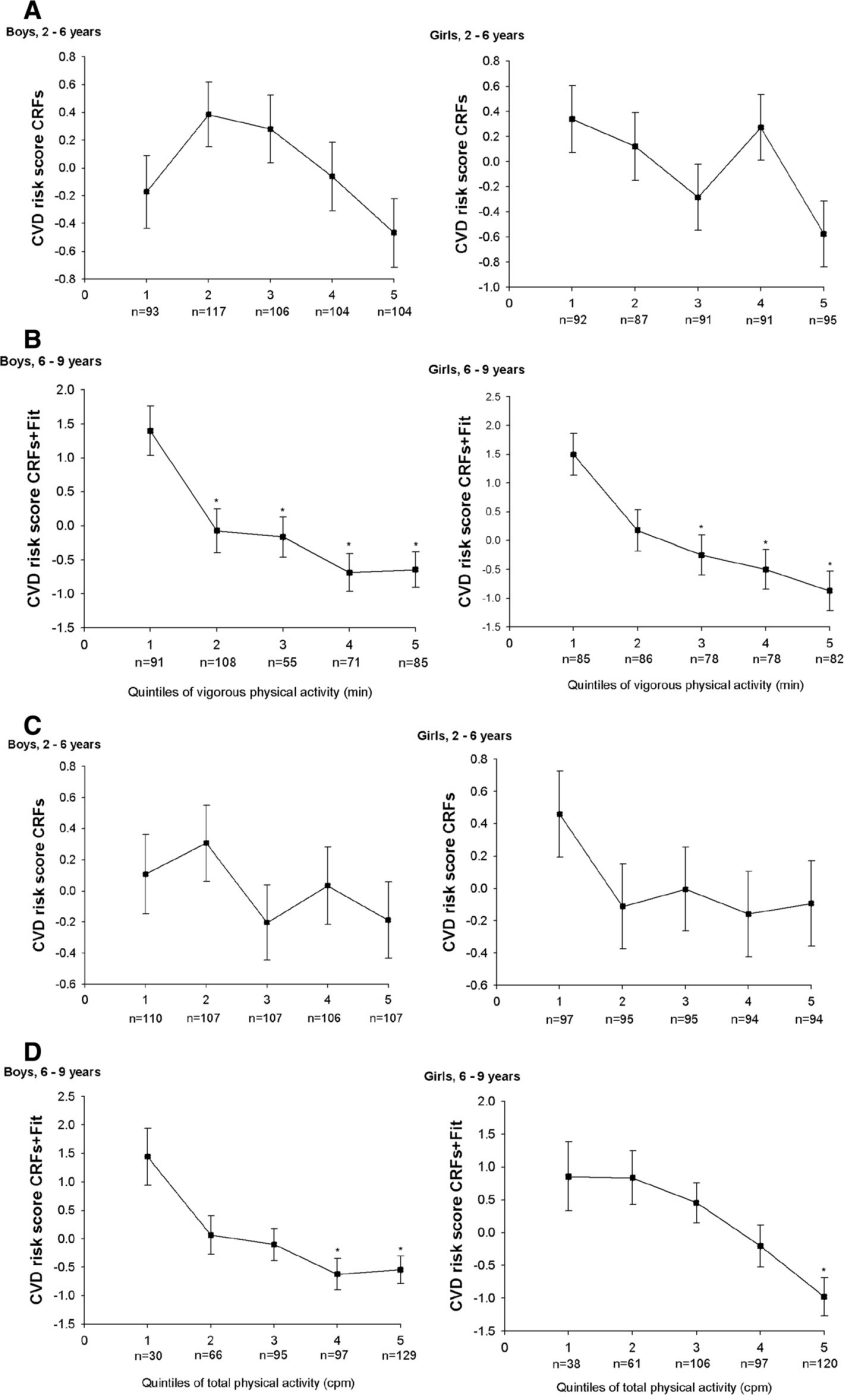
**Cardiovascular disease (CVD) risk score and physical activity (PA) intensities in children aged 2 to 9 years. (A)** Vigorous PA in younger children (2 to 6 years); **(B)** vigorous PA in older children (6 to 9 years); **(C)** total PA in younger children (2 to 6 years); **(D)** total PA in older children (6 to 9 years). Vertical bars show mean ± standard error. *P <0.01 compared to lower quintile. Q5, reference as highest PA levels.

Vigorous PA intensity was the only PA variable that was associated with CVD risk score in younger children. In addition, this was the strongest correlation with CVD risk scores in older children and, consequently, was used in subsequent analyses. Total PA was also used as an overall activity indicator. Figure 1 (A to D) depicts mean Z score in each quintile of PA segregated by age and gender groups. In younger children, non-significant differences in CVD risk score CRFs through quintiles of vigorous (Figure 1A-B) and total PA (Figure 1C-D) were observed. In older children, significantly lower values of CVD risk score CRFs + fit through quintiles of vigorous PA (Figure 1A-B) and total PA (Figure 1C-D) were observed in both genders (all *P* <0.01). Additional analyses using score A instead of score B showed similar results. Additional sensitivity analyses using tertiles instead of quintiles or the PA cut-offs of van Cauwenberghe *et al*. [34] were made, and the results did not change substantially.

### Likelihood for metabolic risk

The risks of having CVD risk score CRFs (dichotomous Z score >1 SD) for PA intensities in children from both age groups are summarized in Table 2. In young boys, the ORs, compared with the most active quintile, were raised in the least active quintile of total PA 2.58 (95% CI 1.07 to 6.18) and in the second quintile for vigorous PA (OR: 2.91; 95% CI 1.25 to 6.82). In older children, the risk of having CVD risk score CRFs decreased with increased activity for all PA intensities. OR for the least active quintiles compared to the most active quintiles of the different PA intensities ranged from 2.69 to 3.82 and 2.32 to 2.95 in boys and girls, respectively (Table 2). When the CVD risk score was calculated using BMI instead of the sum of two skinfold thickness measurements, the results did not change substantially. When sensitivity analyses using tertiles instead of quintiles or other PA cut-offs [34] were used, the results did not change substantially.

**Table 2:** Odds ratios for cardiovascular disease risk score CRFs by quintiles of PA

**Group**	**Quintile**	**Total PA**		**Moderate PA**		**Vigorous PA**		**MVPA**	
		**OR**	**95% CI**	**OR**	**95% CI**	**OR**	**95% CI**	**OR**	**95% CI**
Boys (2 to 6 years)									
n = 524	Q1	**2**.**58**	**1**.**07** to **6**.**18**	1.26	0.55 to 2.88	1.23	0.46 to 3.26	1.39	0.56 to 3.45
	Q2	1.59	0.65 to 3.91	1.27	0.57 to 2.83	**2**.**91**	**1**.**25** to **6**.**82**	2.21	0.95 to 5.15
	Q3	1.31	0.55 to 3.16	1.17	0.51 to 2.66	1.98	0.79 to 4.93	1.98	0.85 to 4.58
	Q4	2.21	0.98 to 4.98	1.23	0.56 to 2.69	2.14	0.89 to 5.15	1.62	0.68 to 3.85
	Q5 (reference)	1		1		1		1	
Girls (2 to 6 years)									
n = 470	Q1	1.03	0.43 to 2.47	0.77	0.32 to 1.85	2.54	0.88 to 7.29	1.23	0.48 to 3.17
	Q2	0.76	0.32 to 1.85	0.80	0.33 to 1.93	2.71	0.96 to 7.70	1.18	0.45 to 3.08
	Q3	0.81	0.34 to 1.90	0.69	0.29 to 1.67	1.68	0.55 to 5.15	1.26	0.50 to 3.13
	Q4	0.72	0.31 to 1.63	0.67	0.28 to 1.62	2.29	0.82 to 6.37	1.20	0.48 to 2.98
	Q5 (reference)	1		1		1		1	
Boys (6 to 9 years)									
n = 1,038	Q1	**3**.**26**	**1**.**74** to **6**.**10**	**3**.**58**	**1**.**95** to **6**.**55**	**3**.**82**	**2**.**05** to **7**.**16**	**3**.**77**	**2**.**04** to **6**.**96**
	Q2	1.70	0.89 to 3.25	1.38	0.75 to 2.53	**2**.**70**	**1**.**45** to **5**.**14**	**1**.**98**	**1**.**04** to **3**.**77**
	Q3	**2**.**00**	**1**.**06** to **3**.**79**	1.29	0.70 to 2.38	**2**.**01**	**1**.**04** to **3**.**89**	1.32	0.67 to 2.59
	Q4	1.10	0.55 to 2.20	0.50	0.24 to 1.06	1.78	0.88 to 3.59	1.23	0.61 to 2.46
	Q5 (reference)	1		1		1		1	
Girls (6 to 9 years)									
n = 987	Q1	1.72	0.90 to 3.30	**2**.**54**	**1**.**33** to **4**.**86**	**2**.**49**	**1**.**37** to **4**.**52**	**2**.**95**	**1**.**55** to **5**.**61**
	Q2	**2**.**47**	**1**.**34** to **4**.**56**	**2**.**78**	**1**.**46** to **5**.**24**	**1**.**90**	**1**.**03** to **3**.**50**	**2**.**70**	**1**.**43** to **5**.**10**
	Q3	**1**.**87**	**1**.**01** to **3**.**45**	**2**.**44**	**1**.**29** to **4**.**62**	1.78	0.94 to 3.39	**2**.**11**	**1**.**11** to **4**.**12**
	Q4	1.31	0.69 to 2.51	1.13	0.56 to 2.28	1.18	0.59 to 2.34	1.27	0.64 to 2.52
	Q5 (reference)	1		1		1		1	

CVD risk score A includes SBP, HOMA-IR, ratio cholesterol/HDL-c, triglycerides and sum of two skinfold measurements. Significant associations are highlighted in bold.

*CRF* CVD risk factor, *CVD* cardiovascular disease, *HDL* high-density lipoprotein, *HOMA*-*IR* homeostasis model assessment, *MVPA* moderate + vigorous PA, *PA* physical activity, *Q* quintile, *Q5* reference as highest PA levels, *SBP* systolic blood pressure.

Since cardiorespiratory fitness data were only available for older children, the logistic regression analyses were repeated for the CVD score CRFs + fit including the reciprocal of the cardiorespiratory fitness scores. The results for both genders are summarized in Table 3. Likelihood ratios for score CRFs + fit were higher than for score CRFs. Children in the first, second and third quintiles had OR for CVD risk score ranging from 2.69 to 5.40 in boys and from 2.85 to 7.05 in girls, relative to the most active quintile. Sensitivity analyses did not change the results substantially. Table 4 summarizes the time-per-day spent at the different PA intensities in the five quintiles of PA, and the cpm of total PA.

**Table 3:** **Odds ratios for cardiovascular disease risk score CRFs** + **fit by quintiles of PA**

**Group**	**Quintile**	**Total PA**		**Moderate PA**		**Vigorous PA**		**MVPA**	
		**OR**	**95% CI**	**OR**	**95% CI**	**OR**	**95% CI**	**OR**	**95% CI**
Boys (6 to 9 years)									
n = 414	Q1	**3**.**36**	**1**.**17** to **9**.**64**	**5**.**40**	**2**.**05** to **14**.**20**	**2**.**69**	**1**.**06** to **6**.**80**	**4**.**36**	**1**.**62** to **11**.**71**
	Q2	1.61	0.61 to 4.21	2.05	0.76 to 5.58	1.65	0.66 to 4.16	2.38	0.91 to 6.23
	Q3	1.91	0.79 to 4.61	2.00	0.75 to 5.31	1.15	0.44 to 2.99	2.06	0.78 to 5.48
	Q4	0.95	0.36 to 2.48	1.16	0.42 to 3.20	0.73	0.26 to 2.04	1.29	0.47 to 3.56
	Q5 (reference)	1		1		1		1	
Girls (6 to 9 years)									
n = 421	Q1	2.20	0.68 to 7.16	**3**.**70**	**1**.**21** to **11**.**29**	**5**.**88**	**2**.**20** to **15**.**76**	**5**.**95**	**1**.**86** to **19**.**05**
	Q2	**4**.**07**	**1**.**66** to **9**.**98**	**4**.**46**	**1**.**69** to **11**.**77**	**2**.**85**	**1**.**02** to **7**.**96**	**7**.**05**	**2**.**47** to **20**.**15**
	Q3	**2**.**94**	**1**.**29** to **6**.**70**	**5**.**34**	**2**.**12** to **13**.**42**	1.87	0.64 to 5.51	**5**.**23**	**1**.**81** to **15**.**13**
	Q4	1.59	0.65 to 3.93	1.88	0.66 to 5.38	1.84	0.63 to 5.36	3.46	1.17 to 10.26
	Q5 (reference)	1		1		1		1	

CVD risk score B includes SBP, HOMA-IR, ratio cholesterol/HDL-c, triglycerides, sum of two skinfolds and cardiorespiratory fitness. Significant associations are highlighted in bold.

*CRF* CVD risk factor, *CVD* cardiovascular disease, *HDL* high-density lipoprotein, *HOMA*-*IR* homeostasis model assessment, *MVPA* moderate + vigorous PA, *PA* physical activity, *Q* quintile, *Q5* reference as highest PA levels, *SBP* systolic blood pressure.

**Table 4:** Time per day spent at the different PA intensities in the five quintiles of PA

**Quintiles by group**	**Total PA**			**Moderate PA**			**Vigorous PA**			**MVPA**		
	**Counts**/**min**	**SD**	**Range**	**Min**/**day**	**SD**	**Range**	**Min**/**day**	**SD**	**Range**	**Min**/**day**	**SD**	**Range**
Boys (2 to 6 years)												
Q1	389.6	59.7	187.2 to 470.9	13.3	4.1	1.3 to 18.7	0.9	0.4	0.2 to 1.5	14.9	4.6	1.3 to 21.3
Q2	525.2	28.2	470.9 to 572.0	22.9	2.2	18.8 to 26.3	2.3	0.4	1.7 to 3.0	25.9	2.4	21.7 to 29.8
Q3	616.4	24.9	572.4 to 659.6	31.0	2.7	26.5 to 35.6	3.9	0.4	3.3 to 4.7	35.3	2.9	30.3 to 39.8
Q4	708.8	31.6	659.8 to 765.8	41.2	3.8	35.7 to 47.7	6.2	1.1	4.8 to 8.5	48.1	4.7	40.3 to 57.2
Q5 (reference)	893.7	97.5	769.0 to 1,239.8	61.1	11.1	48.0 to 115.7	14.7	6.3	8.7 to 37.7	73.2	13.2	57.3 to 141.0
Girls (2 to 6 years)												
Q1	357.3	58.2	173.9 to 428.2	10.1	3.0	14.8 to 21.5	0.9	0.4	0.2 to 1.4	11.3	3.6	3.3 to 16.7
Q2	473.2	24	432.8 to 516.2	18.2	2.1	22.0 to 29.7	2.2	0.4	1.6 to 2.8	21.5	2.5	17.0 to 25.3
Q3	548.5	19.7	516.7 to 582.4	25.7	2.1	29.8 to 38.0	3.9	0.6	3.0 to 4.8	30.0	2.7	25.8 to 34.3
Q4	637.4	32.4	584.1 to 693.4	33.7	2.4	38.3 to 77.7	6.1	0.8	5.0 to 7.8	39.9	3.0	35.0 to 45.3
Q5 (reference)	806.7	103	694.8 to 1,210.6	47.9	9.2	24.7 to 34.3	12.2	6.0	8.0 to 58.3	57.8	11.7	45.3 to 107.0
Boys (6 to 9 years)												
Q1	383.5	60.6	165.4 to 461.4	17.5	5.2	3.0 to 24.3	1.6	0.7	0.3 to 2.8	20.3	6.2	3.0 to 28.5
Q2	507.6	24.7	461.4 to 548.2	29.7	2.8	24.7 to 34.3	3.8	0.6	3.0 to 4.9	34.8	3.4	28.7 to 40.3
Q3	594.4	24.3	548.6 to 635.8	38.2	2.4	34.3 to 42.6	6.1	0.8	5.0 to 7.6	45.4	3.1	40.6 to 50.8
Q4	690.3	34.4	635.9 to 752.7	48.2	3.5	42.7 to 54.5	9.9	1.3	7.8 to 12.3	57.4	4.5	51.0 to 65.8
Q5 (reference)	871.1	99.9	753.2 to 1,258.7	69.3	12.3	54.8 to 115.0	18.3	5.8	12.3 to 42.0	84.6	15.4	66.3 to 137.5
Girls (6 to 9 years)												
Q1	354.3	52.6	123.1 to 414.0	12.6	3.7	1.3 to 17.8	1.5	0.6	0.3 to 2.4	15.0	4.6	1.3 to 21.8
Q2	459.1	24.5	414.7 to 499.5	21.3	2.0	18.3 to 24.6	3.3	0.5	2.6 to 4.2	26.0	2.6	21.8 to 30.0
Q3	538.6	21.9	499.8 to 575.7	28.2	2.1	24.8 to 31.8	5.3	0.7	4.3 to 6.5	34.3	2.4	30.3 to 38.7
Q4	621.0	28.9	575.7 to 677.9	36.2	2.8	32.0 to 41.3	8.5	1.1	6.7 to 10.7	45.0	3.3	39.2 to 51.3
Q5 (reference)	789.9	97.7	680.7 to 1,198.9	52.5	9.4	41.7 to 103.5	17.3	5.9	11.3 to 46.3	66.4	13.1	53.1 to 131.5

*PA* physical activity, *MVPA* moderate + vigorous PA, *Q* quintile, *Q5* reference as highest PA levels.

## Discussion

The main findings of the study were the inverse associations between PA and clustered CVD risk factor scores. The risk was raised in the first to third quintiles of PA for older children compared to the most active quintile, while in younger children some inverse association were found only for boys, but not sufficiently consistent. The time spent at MVPA in the fifth quintile was a mean of 85 minutes and 66 minutes in older children (boys and girls, respectively); therefore, the current recommendation for PA of at least 60 min/day of at least moderate intensity in order to avoid the negative consequences of clustering of risk factors could be appropriate for girls but might be a slight underestimate for boys.

### Comparison with other studies

Our findings concur with others that had observed inverse associations between PA and CVD risk factors [9-16]. However, most of these studies had been conducted in children at the ages of 9 and 15 years. Our study observed this inverse relationship between objectively-measured PA and CVD risk score in children aged 2 to 9 years who constitute a less well investigated age range, establishing that there is only consistent evidence for children aged 6 to 9 years. In addition, our results provided more relevant gender-specific data on the strength of association and the PA intensities. Moreover, Butte *et al*. performed the only study with children aged of 4 to 19 years, but failed to show a clear association between PA and CVD risk factors [11]. In a first approach the correlations were significant, although small variances were seen, which could be partially due to the overall low PA levels found in this sample. Despite that, in the present study an inverse relationship between PA and CVD risk score was found consistently in older boys and girls (6 to 9 years). The weaker associations observed exclusively in younger boys may be due to these CVD risk factors not being manifest as yet in this age group of particularly young children (2 to 6 years); this is in accord with other authors who failed to found any association in younger children (6 years old) [1,17]. Moreover, the relatively healthy sample from this study, as well as the impossibility to use the score with cardiorespiratory fitness, could hamper the sensitivity in detecting associations [35]. However, more studies should look at this age range in order to corroborate that assumption. Non-concordance between studies could be due to methodological differences such as sample size (lower in others vs current study), ethnic origin, age range, and stratification of the data for analysis.

The present study focused on children aged 2 to 9 years. This is a younger age group than the age groups included in those studies on which previous recommendations had been based (6 to 17 years) [9,18-23]. Since the consensus is that PA requirements should be age specific [7] our data analyses were performed in two age groups: 2 to 6 and 6 to 9 years of age. This enables, for the first time, separate recommendations to be formulated for children <6 years of age, and for those who are older. Nevertheless, the lack of consistency in the younger group make it necessary to be cautious when interpreting the levels of PA in younger children, as it should not be used for recommendation but only for description. Our study observed that, in girls from the older age group, the current recommendation [23] of PA of at least 60 min/day of at least moderate intensity could be enough to prevent the negative consequences of CVD risk factor scores. However, in boys from the same age group, 85 minutes (rather than 60 minutes) could be a more appropriate threshold to ensure a lower CVD risk factor score and this value is close to the 90 minutes daily PA suggested by Andersen *et al*. [9]. Further, an important finding in our study is that the mean time spent on vigorous PA in older children should be around 20 min/day. Our findings regarding time spent at vigorous PA in order to pre-empt potential CVD risk are in agreement with other studies, which observed that a similar amount of vigorous PA can discriminate between normal weight and overweight [36] while being associated with better bone mineral content [37]. To the best of our knowledge, ours is the first study that has analyzed the association between objectively-measured PA and clustered CVD risk factors in a large sample of children from 2 to 9 years, as well as providing gender-specific recommendation for children aged 6 to 9 years.

### Strengths and limitations

The strengths of the present study are the availability of standardized measures of objective PA, insulin resistance and other CVD risk factors, as well as cardiorespiratory fitness. Further, having a well balanced gender distribution within a large heterogeneous sample of young children from eight European countries provides an excellent opportunity to derive gender-specific data. To date, studies regarding the association between PA and CVD risk factors (as well as the current guidelines for PA) have been focused mainly on older children. Our study, instead, covers ages from 2 to 9 years. These aspects are of interest for public health since they provide new insights into PA needs and recommendations for younger children that may used by physicians and other healthcare workers.

The present study has several limitations, however. The cross-sectional nature of the study precludes determining any causality in the findings. The overall healthy sample, with only 15% of children above 1 SD of the CVD score, could limit the interpretation of the present results, particularly in a less healthy population. Only interventional studies with exercise could establish whether or not these specific recommendations are effective in reducing cardiovascular risk, and our findings have not established the efficacy of these recommendations. More randomized controlled trials and prospective studies are needed to focus on improving CVD risk factor status through increasing the volume and intensity of PA and differentiating by age range.

## Conclusions

PA is important to prevent a clustering of risk factors in young children aged 6 to 9 years. In clinical settings, practitioners should recommend that, in girls, the current guidelines of at least 60 min/day of PA of at least moderate intensity could be enough, but around 20 of these minutes should be of vigorous intensity. However in boys, 85 min/day MVPA including around 20 minutes vigorous PA could be necessary to prevent the negative consequences ascribed to clustering of risk factors. In younger children aged 2 to 6 years it seems that this role of PA is less consistent than in older children based on the low numbers of significant associations, although indications of some influence of PA as a preventive tool were observed in such young children. The assessment of how changes in PA volume and intensity can causally affect clustering of CVD risk factors in young children remains to be properly explored. Future interventions are needed to identify how much increase in PA intensity and volume would be required to improve CVD risk factor status.

## Abbreviations

ANCOVA: Analysis of covariance; BMI: Body mass index; CVD: Cardiovascular disease; CRFs: Continuous score clustering CVD risk factors (SBP, TG, TC/HDL-c ratio, HOMA-IR, and sum of two skinfolds); CRFs + fit: CVD risk score containing the score CRFs + the cardiorespiratory fitness; DBP: Diastolic blood pressure; HDL-c: High-density lipoprotein cholesterol; HOMA-IR: Homeostasis model assessment of insulin resistance; IDEFICS: ‘Identification and prevention of Dietary- and lifestyle-induced health EFfects In Children and infantS’; MVPA: Moderate and vigorous PA; PA: Physical activity; SBP: Systolic blood pressure; SES: Socioeconomic status; TC: Total cholesterol; TG: Triglycerides.

## Competing interests

We have employed the services of a professional medical writer, but only for the purposes of editorial assistance and English language verification. The payment for these services has been internal and does not involve sources with any vested interests in the findings of the study. The authors declare that they have no competing interests.

## Authors’ contributions

DJ-P, YP and LAM contributed to the concept and design of the study. DJ-P, WA, HP, AS, LI, CH, DM, SDH, YP and LAM contributed to the conduct of the study. DJ-P, KK, PB and LAM contributed to the analysis and interpretation of data. DJ-P, KK, PB, YP, and LAM contributed to drafting the manuscript. DJ-P, WA, HP, CH, AS, LI, DM, SDH, YP and LAM critically reviewed the manuscript. DJ-P is the guarantor of this work and, as such, had full access to all the data in the study and takes responsibility for the integrity of the data and the accuracy of the data analysis. All authors read and approved the final manuscript.

## Authors’ information

All the authors take responsibility for all aspects of the reliability and freedom from bias of the data presented and their discussed interpretation.
